# Exploring Interleukin Levels in Type 1 Diabetes and Periodontitis: A Review with a Focus on Childhood

**DOI:** 10.3390/children11020238

**Published:** 2024-02-13

**Authors:** Silvia D’Agostino, Giulia Valentini, Marco Dolci

**Affiliations:** 1 Complex Unit of Odontostomatology, Interdisciplinary Department of Medicine, University of Bari “Aldo Moro”, 70124 Bari, Italy; 2 Department of Medical, Oral and Biotechnological Sciences, University G. d’Annunzio, 66100 Chieti, Italy; giuxval@gmail.com (G.V.); marco.dolci@unich.it (M.D.)

**Keywords:** childhood, interleukins, periodontitis, peripheral inflammation, single-nucleotide polymorphisms, systemic inflammation, type 1 diabetes

## Abstract

Diabetes can trigger an increase in cytokine levels leading to the production of C-reactive protein and fibrinogen. These molecules promote subclinical inflammation, causing the expression of adhesive molecules and endothelial dysfunction. Despite the lack of a comprehensive panel for single-nucleotide polymorphisms (SNPs) for interleukins associated with type 1 diabetes mellitus (T1DM), understanding the inflammatory role of SNPs is crucial because periodontitis, the sixth complication of diabetes, is influenced via these genetic variations. This review focuses on the interleukin levels in T1DM patients with and without periodontitis, with a particular focus on childhood and on SNPs when reported. A search of PubMed and Scopus identified 21 relevant studies from the past five years. Several ILs were analyzed, emphasizing that T1DM still needs to be thoroughly explored regarding an IL polymorphisms panel; however, the last five years have led to the increased independence of this condition, causing autonomous inflammatory effects, which require further investigation. The periodontitis and T1DM association in children and adolescents represents a severe gap in the literature that should be filled; this scarce presence of studies serves as motivation for further clinical research.

## 1. Introduction

Type 1 diabetes mellitus (T1DM) is a chronic condition in which the pancreas produces little or no insulin by itself—it is a complex autoimmune disease with genetic implications defined by T-cell-mediated destruction of pancreatic β cells leading to uncontrolled hyperglycemia and insulin dependence [1]. It occurs mainly in childhood and adolescence, although cases in adulthood are not so uncommon, which is known as latent autoimmune diabetes (LAD) [2]. People with LAD may have a residual β-cell function, and diabetes onset could be slow and free of insulin dependence for years after the diagnosis. Two different groups can be recognized: The first group is Autoimmune Diabetes Mellitus, which is caused by beta-cell destruction by antibodies and mostly affects subjects in their infancy and adolescence. The other group is represented by Asian and African ethnicities, with a still controversial pathogenesis and the presence of hyperglycemia but no autoimmune dysfunction. The development of T1DM is the result of an elaborate interplay of multiple genetic and environmental factors that are still poorly recognized, although evidence seems to point out that the environment plays a role in T1DM expression, as demonstrated by different expressions in genetically similar populations [3]. Chronic low-grade inflammation and environmental factors seem to play a crucial role in the development of this pathology. Chronic low-grade inflammation is a state of persistent inflammation that is not caused by an infection or injury. It is thought to be involved in the development of a number of diseases, including T1DM [3].

Environmental factors are factors that are outside of the body, such as viruses, bacteria, and chemicals. They can also include determinants like diet, exercise, and stress. People with T1DM have higher levels of pro-inflammatory cytokines; however, the exact way in which chronic low-grade inflammation and environmental factors contribute to the development of T1DM is not fully understood. It is thought that they may both contribute to damage the cells in the pancreas that produce insulin. The damage to these cells leads to the development of T1DM, which is a chronic condition that requires lifelong treatment. In 2021, T1DM involved 8.4 million individuals, of which 18% (1.5 million) were younger than 20 years old, meaning that about 1 in 6 people with T1DM are a child or adolescent [4]. Human leukocyte antigens (HLAs) are a group of genes that are involved in the immune system. HLA class II genes are particularly important in the recognition of foreign antigens, such as those found on bacteria and viruses. People with certain HLA class II genes are more likely to develop T1DM. This is because these genes can help the immune system to mistakenly attack the cells in the pancreas that produce insulin. The HLA class II genes that are most strongly associated with T1DM are the HLA-DR and HLA-DQ genes. People who carry certain variants of these genes are more likely to develop T1DM than people who do not carry these variants. The exact way in which HLA class II genes contribute to the development of T1DM is not fully understood; however, it is thought that they may play a role in the activation of T cells, which are a type of white blood cell that is involved in the immune system. T cells that are activated by HLA class II genes can mistakenly attack the cells in the pancreas that produce insulin. This can lead to the destruction of these cells and the development of T1DM. The identification of HLA class II genes that are associated with T1DM has helped to improve our understanding of the disease. This knowledge can be used to develop new diagnostic tests and treatments for T1DM [5]. Interleukin-1α (IL-1α), a well-known pro-inflammatory cytokine, plays a relevant role in the immune response and inflammatory regulation. For this reason, single-nucleotide polymorphisms (SNPs) could lead to different proteins with an influence on immune response and, subsequently, with measures of glucose homeostasis and diabetes [6]. Recent advances in research have highlighted that patients affected by T1DM have an altered IL expression. Some studies have shown that people with T1DM have certain polymorphisms, or variations, in the genes that code for ILs. These polymorphisms may make people more likely to develop T1DM, or they may make the disease more severe. While it is true that a single genetic polymorphism is not directly correlated with autoimmune reactions, the relationship between genetics and autoimmunity is more nuanced. Therefore, it is not accurate to say there is no correlation between genetic polymorphisms and autoimmune reactions; it is a complex interplay, and attributing a direct cause-and-effect relationship is not always possible. Periodontitis (P), a pathology characterized by the progressive loss of periodontal tissue with potential tooth loss, has been demonstrated to be in tight conjunction with DM. P is considered to be the sixth complication of DM [7]. Although systemic low-grade inflammation, as an expression of IL polymorphism, seems to play a role in P development, few studies in the literature have investigated this aspect in recent years. In the recent past, few studies have been conducted in order to assess if IL polymorphism could be considered a preventive tool to assess the risk of developing P or to assess its progression. One of these studies evaluated the presence of IL-1 SNPs in association with the presence of other risk factors, it aimed to stratify the population into different ranges of risk of developing P and to evaluate the effectiveness of preventive care. The aim of that paper was to assess where resources for preventive treatments could be better targeted to achieve better results in terms of prevention and care [8]. On the other hand, a recent study about the relation of IL-8 polymorphism and P revealed that, although the relation between IL-8 and DM has been clearly assessed, a relationship between the circulation of periodontal bacteria and hematic IL-8 was not confirmed; therefore, the relationship between P and IL-8 polymorphism, although advocated, was not validated [9]. In another study, the T1DM control and the “red complex” occurrence seemed to be partially influenced by IL-17A in both diabetic patients with P and non-diabetic patients with P [10]. The aim of the present study was to evaluate the relationships between IL levels, T1DM, and P; the secondary outcome was to highlight the connection between SNPs and childhood.

## 2. Materials and Methods

A systematic review was conducted using the Preferred Reporting Items for Systematic Reviews and Meta-Analyses (PRISMA) guidelines for systematic reviews and meta-analyses [11] and registered on PROSPERO—International Prospective Register of Systematic Reviews—with ID code CRD42022385079 [12].

### 2.1. The Literature Search

The purpose of the literature search was to identify relevant studies investigating the linking between T1DM and IL polymorphisms in the last five years, a comprehensive search of PubMed and Scopus, using the Patient/Population/Problem, Intervention, Comparison, and Outcome (PICO) format, was conducted.

- Population: humans and animals;
- Intervention: IL polymorphisms in healthy subjects;
- Comparator: IL polymorphisms in T1DM patients/animals;
- Outcomes: correlation between IL polymorphisms and T1DM.

The following MeSH were used: interleukins; periodontitis; single-nucleotide polymorphisms; type 1 diabetes mellitus.

### 2.2. Eligibility Criteria

The inclusion criteria were as follows: all studies analyzing the interleukin levels and the variety of polymorphisms among interleukins linked with T1DM, in humans and animals.

The exclusion criteria were as follows: research about polymorphisms of other cytokines; papers about the links between interleukins and other pathological conditions; systematic reviews; meta-analyses; editorials; abstracts.

### 2.3. Data Extraction

Studies were screened by two reviewers independently, and a matrix of relevant data was produced. Disagreements were solved by consensus with a third reviewer. Data extraction included general details relating to the characteristics of the studies (e.g., author, year of publication, sources of funding) and specific details about the type of interleukins and their polymorphisms.

### 2.4. Quality Assessment

As shown in Table 1, data quality assessment of included studies was rated using the Joanna Briggs Institute (JBI) Critical Appraisal Checklist for Cohort Studies [13]. Several fields impacting the level of certainty were evaluated using this tool, such as publication bias, heterogeneity, reliability of methods employed to measure outcomes, study design limitations, reverse causality, and imprecision. Additionally, it takes into account domains that can enhance the level of certainty, such as the appropriateness of statistical analysis, a large magnitude effect, and opposing plausible residual bias or confounding. Potential discrepancies were discussed with a third author. A qualitative description of the characteristics of the included studies as well as a narrative data synthesis were performed (Table A1).

## 3. Results

### 3.1. Overall Scenario

**Figure 1:**
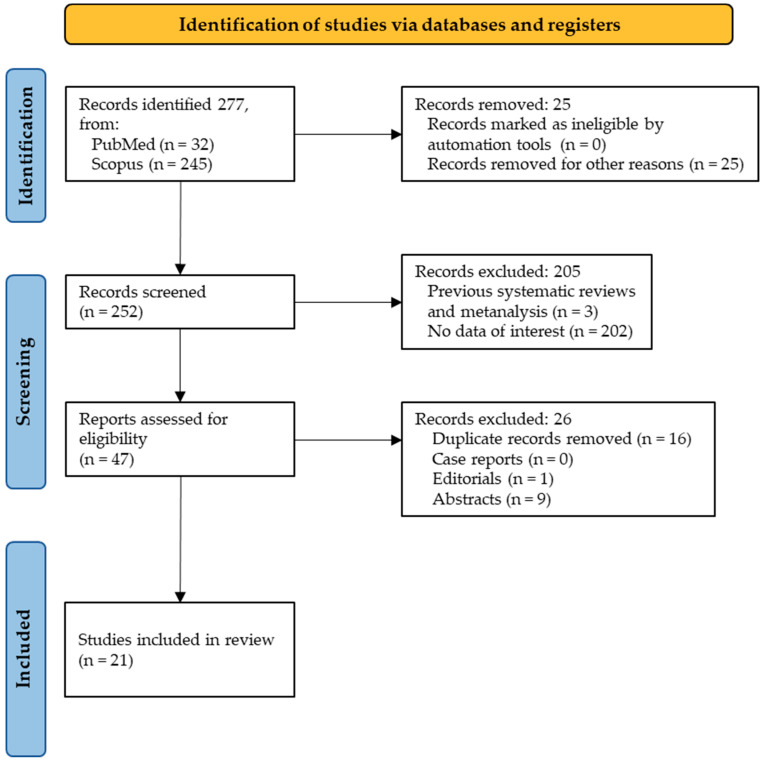
PRISMA flowchart.

The initial search provided a total of 277 studies: 32 from PubMed and 245 from Scopus. No studies were removed for being marked as ineligible by automation tools, while 25 studies were removed for other reasons, for example, for the study of other diseases in addition to diabetes, or for the analyses of other cytokines. A total of 252 studies were included in the screening phase, and a total of 205 studies were removed because of a lack of data of interest (202) or because they were a systematic review with or without metanalysis (3). Eligibility was assigned to 47 studies, from which 16 were removed for being duplicates, 9 for being abstracts, and 1 for being an editorial. Finally, a total of 21 studies were involved in the inclusion phase (Figure 1).

### 3.2. Detailed Results

None of the included articles concerned animals. Regarding the population age, 38.1% (8/21) of studies refer to adults, 23.8% (5/21) refer to children and adolescents, 14.3% (3/21) refer to adolescents, 9.5% (2/21) refer to children, 9.5% (2/21) refer to all ages, and, finally, 4.8% (1/21) refer to children and adults. The most investigated ethnicity was the German population in 19% (4/21) of studies, followed by the Euro-Brazilian people in 14.3% (3/21) of studies. Other ethnic groups such as Chinese, Saudi, Egyptian, and Czech appeared each in 9.5% (2/21) of reports. The least-investigated clusters were Iranian, Indian, Thai, Scandinavian, Emirati, and Brazilian in 4.8% (1/21) of cases. Interleukins analyzed in descending order were IL-6, IL-2, IL-7, IL-1, IL-10, IL-17, IL-4, IL-8, IL-18, and finally IL-12. The most-used technique to examine these specific cytokines was the Polymerase Chain Reaction (PCR), more rarely Next-Generation Sequencing (NGS) and mass spectrometry. All included studies used peripheral blood samples to perform these tests. Only 14.3% (3/21) of studies discuss periodontitis. Due to considerable heterogeneity in the polymorphism regions among several interleukin measures, a formal meta-analysis was not carried out.

The results of the JBI Critical Appraisal Checklist for Cohort Studies are shown in Table 1. None of the studies analyzed were excluded by this quality assessment.

All the included studies are presented in Table 2, including the authors, year, population, nationality, interleukin(s) and number of the polymorphism, and type of analysis. A brief narrative summary is shown in Table A1.

## 4. Discussion

A systematic review following the PRISMA flowchart was performed in order to assess the relationships between IL SNPs and T1DM, as well as with periodontitis.

IL-1 and its SNPs were investigated by Li J. et al. [22], more specifically, the authors of the study measured the concentration of IL-1β, a pro-inflammatory cytokine, in the blood of patients with T1DM and healthy controls. They found that the concentration of IL-1β was significantly higher in patients with T1DM compared to healthy controls. This suggests that IL-1β may play a role in the development of T1DM. Tangjittipokin W. et al. [24] assessed that IL1B SNPs are associated with T1DM susceptibility. Variations in the IL2RA gene could be a risk factor for vascular complications in people with T1DM [26]. The IL2RA gene codes for the IL2 receptor alpha (IL-2Rα). IL-2Rα is a key regulator of the immune system, and it is involved in the triggering of T cells that play a role in fighting infection.

Some studies have shown that people with certain variants in the IL2RA gene are more likely to develop vascular complications, such as retinopathy, nephropathy, and cardiovascular disease, if they have T1DM. This suggests that IL2RA gene variants may increase the risk of developing these complications. The exact mechanism by which IL2RA gene variants increase the risk of vascular complications is not fully understood; however, it is thought that these variants may alter the way that T cells respond to certain antigens. This could lead to the activation of T cells that attack the cells in the pancreas that produce insulin, or it could lead to the production of pro-inflammatory cytokines that contribute to the development of vascular complications.

More research is needed to understand the role of IL2RA gene variants in the development of vascular complications in people with T1DM; however, recent findings suggest that IL2RA gene variants may be a target for the development of new treatments for these complications, and could confer risk alleles for T1DM among the Emirati population [27]. In a study published in 2021, Osman A. E. and colleagues [23] investigated the levels of ILs in patients with T1DM and healthy controls. They found that the levels of IL-1A/B, IL-2, and IL-12 were significantly higher in patients with T1DM in contrast to healthy controls.

The authors of the study hypothesized that these elevated levels of ILs may contribute to the development and progression of T1DM. They also suggested that ILs could be a potential target for the development of new treatments for the disease.

The study by Osman A. E. et al. [23] is one of several studies that have shown that ILs may play a role in T1DM. SNPs are variations in DNA that can occur at a single point in the genome. Some SNPs have been linked to an increased risk of developing certain diseases, including type 1 diabetes mellitus (T1DM). One SNP that has been linked to T1DM is rs2070874, which is located in the IL-4 gene. The IL-4 gene codes for a protein called interleukin-4 (IL-4), which is a cytokine that influences the immune system. People who carry the rs2070874 SNP are more likely to produce lower levels of IL-4. This is because the SNP changes the DNA sequence in the IL-4 gene, which makes it less likely that the gene will be able to produce IL-4. Low levels of IL-4 have been linked to an increased risk of developing T1DM. This is because IL-4 is thought to serve as a protection of the body from developing autoimmune diseases. When IL-4 levels are low, the body is more likely to attack its own cells, which can lead to the development of T1DM. The risk of developing T1DM is especially high in young individuals who carry the rs2070874 SNP and who also carry vulnerable HLA alleles/haplotypes. HLA alleles/haplotypes are genes that are involved in the immune system. People who carry vulnerable HLA alleles/haplotypes are more likely to develop T1DM, even if they do not carry the rs2070874 SNP. These findings suggest that the rs2070874 SNP may be a useful marker for identifying people who are at increased risk of developing T1DM. This information could be used to develop early intervention strategies that could help prevent the development of the disease. Therefore, SNP associated with low production of IL-4 increases the risk of T1DM in young individuals carrying vulnerable HLA alleles/haplotypes [16].

IL-6 was well-investigated by Haghnazari L. et al. [19], assessing that the G allele of SNP rs1042522 encoding the TP53 gene for IL-6 increases the risk of developing DM in an Iranian population. At the same time, IL-6R rs2228145 was associated with T1DM development in adulthood [31].

SNPs for IL-7 in T1DM patients were investigated in several valuable works [14,20,31,32]. Hoffman M. et al. [14] revealed higher sIL-7R serum concentrations at T1DM onset and decreasing levels during therapy, whereas IL-7 was only higher in long-term patients compared to controls. Hehenkamp P. et al. [20] elucidated that T1DM monocytes have an impaired IL-7 response and lower IL-7R expression. According to Lundtoft C. et al. [30], IL-7Rα variants may contribute to disease susceptibility against T1DM. Finally, Seyfarth J. et al. [32] pointed out that only T1DM children with the protective haplotype had lower IL-7 serum levels.

Regarding IL-10, El Helaly R.M. et al. [18] stated that the AA genotype and A allele of IL-10 rs1518111 SNP could be linked to an increased risk for T1DM among Egyptian children. With reference to IL-17, increased serum IL-17A is a risk factor for autoimmune T1DM [15].

Finally, IL-18 has a controversial role because the IL-18 gene-promoter polymorphisms might be associated with susceptibility to T1DM in Egyptian children [18] and with T1DM age onset [29]; however, at the same time, according to Campos L. P. et al. [26], IL-18 polymorphisms were not associated with T1DM onset in children or adults in this population. In addition, Al-Lahham Y. et al. [33] also found that IL-18 rs187238 was not associated with T1DM in a Euro-Brazilian population.

The burden of P is investigated only in a few studies in the present review. In particular, Kumar S. et al. [21] found out that the IL-17A polymorphism was not associated with an increased risk for P in T1DM patients. In a similar way, Borilova Linhartova P. et al. [29] considered the variability in the IL-1B and IL-1RN genes as a possible factor in susceptibility to T1DM and P, although the single variants of these polymorphisms are not crucial for protein production. The same authors also explored the role of IL-8, concluding that IL-8 levels are not influenced by chronic P and that patients with T1DM and P had higher circulating IL-8 levels as opposed to healthy controls with P and non-periodontitis subjects [9]. It has to be stressed that all of these studies about P referred to adults, while childhood and adolescence seemed to be left out. While research suggests elevated IL concentrations in T1DM, the genetics underlying this connection remain unclear and contested. Despite the numerous studies included in this review, a definitive explanation for the role of IL polymorphisms in T1DM’s inflammatory processes is yet to be established, especially in childhood and adolescence. While periodontitis might contribute to increased IL-8 production, existing studies suggest that specific interleukin SNPs (like IL-1B and IL-17) are not significant risk factors for both T1DM and periodontitis development. Further clinical trials are crucial to elucidate the role of such SNPs in T1DM pathogenesis, especially in the context of periodontitis presence or absence.

### Limitations of the Study

A crucial factor of our review was the challenge to overcome study heterogeneity in terms of methods of interleukin analysis and direct or indirect correlation of T1DM and its complications. Sometimes, the authors left out the specific technique of analysis, creating an important bias that must be kept in mind. Another severe bias is represented by the lack of data exploring both T1DM and periodontitis. Finally, other confounding factors were represented by the existence of several polymorphisms detected for the same interleukin.

## 5. Conclusions

To the best of the authors’ knowledge, IL SNPs still represent a controversial field in the understanding of inflammation aspects of T1DM. Most of the studies included in this review pointed out how higher IL concentrations are present in this specific population but a univocal explanation about the genetic characterization is not completely available.

It seems that P could be an adjunctive factor for the production of IL-8; however, SNPs of specific interleukins (IL-1B, IL-17) do not qualify as risk factors in the susceptibility for T1DM and P. More clinical trials are needed on this topic in order to clarify the pathogenetic mechanisms of SNPs in T1DM genesis in patients with and without P. Despite the incomplete knowledge about the full spectrum of interleukin polymorphisms in T1DM, research over the past five years has identified distinct inflammatory pathways that are dependent on unknown factors, requiring more extensive study. The P and T1DM association in children and adolescents represents a severe gap in the literature—there are only three studies available on this—and should be filled; additionally, the scarce presence of studies about adults serves as a motivation for further clinical research.

## Figures and Tables

**Table 1:** The Joanna Briggs Institute (JBI) Critical Appraisal Checklist for Cohort Studies [13]. Q1: were the two groups similar and recruited from the same population? Q2: were the exposures measured similarly to assign people to both exposed and unexposed groups? Q3: was the exposure measured in a valid and reliable way? Q4: were confounding factors identified? Q5: were strategies to deal with confounding factors stated? Q6: were the groups/participants free of the outcome at the start of the study (or at the moment of exposure)? Q7: were the outcomes measured in a valid and reliable way? Q8: was the follow-up time reported and sufficient to be long enough for outcomes to occur? Q9: was follow-up complete, and if not, were the reasons to loss to follow-up described and explored? Q10: were strategies to address incomplete follow-up utilized? Q11: was appropriate statistical analysis used? Y: yes; N: no; U: unclear; Studies were excluded when ≥3 criteria were not met.

Authors/Year	Q1	Q2	Q3	Q4	Q5	Q6	Q7	Q8	Q9	Q10	Q11
Hoffman M. et al., 2022 [14]	Y	Y	Y	N	Y	N	Y	Y	U	Y	Y
Li J. et al., 2022 [15]	Y	Y	Y	Y	Y	Y	N	Y	Y	U	Y
Osman A. E. et al., 2022 [16]	Y	Y	U	Y	Y	Y	Y	Y	Y	Y	Y
Ali Y. et al., 2021 [17]	Y	Y	Y	Y	N	Y	U	Y	Y	Y	Y
El Helaly R.M. et al., 2021 [18]	Y	U	Y	Y	Y	Y	Y	Y	N	Y	Y
Haghnazari L. et al., 2021 [19]	Y	Y	Y	Y	Y	U	Y	Y	Y	Y	Y
Hehenkamp P. et al., 2021 [20]	Y	Y	N	Y	Y	Y	Y	U	Y	Y	Y
Kumar S. et al., 2021 [21]	N	Y	Y	Y	U	Y	Y	Y	Y	Y	Y
Li J. et al., 2021 [22]	Y	Y	N	Y	Y	Y	Y	Y	Y	Y	Y
Osman A. E. et al., 2021 [23]	Y	Y	Y	Y	N	Y	Y	Y	Y	Y	Y
Tangjittipokin W. et al., 2021 [24]	Y	Y	Y	Y	Y	Y	Y	Y	U	U	Y
Campos L. P. et al., 2020 [25]	Y	Y	Y	Y	Y	Y	U	Y	Y	Y	Y
Keindl M. et al., 2020 [26]	N	Y	Y	Y	Y	Y	Y	N	Y	Y	Y
Sharma C. et al., 2020 [27]	U	Y	Y	Y	Y	Y	Y	Y	Y	Y	Y
Boechat-Fernandes A. et al., 2019 [28]	Y	Y	Y	Y	Y	U	Y	Y	Y	Y	Y
Borilova Linhartova P. et al., 2019 [29]	Y	Y	Y	Y	N	Y	Y	Y	Y	Y	Y
Campos L. P. et al., 2019 [30]	Y	Y	Y	Y	Y	Y	Y	Y	Y	N	Y
Lundtoft C. et al., 2019 [31]	Y	Y	Y	Y	Y	N	Y	Y	Y	Y	Y
Borilova Linhartova P. et al., 2018 [9]	Y	Y	Y	U	Y	Y	Y	Y	Y	Y	Y
Seyfarth J. et al., 2018 [32]	Y	Y	Y	Y	Y	Y	Y	U	Y	Y	Y
Al-Lahham Y. et al., 2017 [33]	Y	N	Y	Y	Y	N	Y	Y	Y	Y	Y

**Table 2:** Keys results. Children up to 10 years. Adolescents up to 18 years. Adults were 18 years and over. NGS: Next-Generation Sequencing. PCR: Polymerase Chain Reaction. sIL-2R: soluble IL-2 receptor. IL-2RA: IL-2 Receptor Alpha.

Authors/Year	Population (Age)/Ethnicity	IL/#Polymorphism	Analysis
Hoffman M. et al., 2022 [14]	349 (Children/adolescents)/German	IL-7/2	NGS
Li J. et al., 2022 [15]	270 (Adults)/Chinese	IL-6, IL-17(A-F)/7	PCR
Osman A. E. et al., 2022 [16]	371 (All ages)/Saudi	IL-4, IL-10/5	PCR
Ali Y. et al., 2021 [17]	218 (Children)/Egyptian	IL-6, IL-18/3	PCR
El Helaly R.M. et al., 2021 [18]	230 (Children/adolescents)/Egyptian	IL-10/2	PCR
Haghnazari L. et al., 2021 [19]	136 (Adults)/Iranian	IL-6/1	PCR
Hehenkamp P. et al., 2021 [20]	40 (Children)/German	IL-7/1	PCR
* Kumar S. et al., 2021 [21]	90 (Adults)/Indian	IL-17A/1	PCR
Li J. et al., 2021 [22]	1092 (Adolescents)/Chinese	IL-1B/2	Mass spectometry
Osman A. E. et al., 2021 [23]	328 (All ages)/Saudi	IL-1(A, B), IL-2, IL-12/5	PCR
Tangjittipokin W. et al., 2021 [24]	200 (Children/adolescents)/Thai	IL-2, IL-4. IL-6, IL-10, IL-13, IL-17A/6	PCR
Campos L. P. et al., 2020 [25]	611 (Children/adults)/Euro-Brazilian	IL-18/1	PCR
Keindl M. et al., 2020 [26]	79 (Adults)/Scandinavian	sIL-2R/68	NGS
Sharma C. et al., 2020 [27]	310 (Adolescents/adults)/Emiratis	IL-2RA/1	PCR
Boechat-Fernandes A. et al., 2019 [28]	1101 (Adolescents)/Brazilian	IL-12B, IL-18/3	PCR
* Borilova Linhartova P. et al., 2019 [29]	659 (Adults)/Czech	IL-1/2	PCR
Campos L. P. et al., 2019 [30]	291 (Adults)/Euro-Brazilian	IL-6, IL-6R/2	PCR
Lundtoft C. et al., 2019 [31]	301 (Children/adolescents)/German	IL-7RA/2	PCR
* Borilova Linhartova P. et al., 2018 [9]	109 (Adults)/Czech	IL-8, IL-8R/2	PCR
Seyfarth J. et al., 2018 [32]	301 (Adolescents)/German	IL-7RA, sIL-7R/2	PCR
Al-Lahham Y. et al., 2017 [33]	280 (Adults)/Euro-Brazilian	IL-18/1	PCR

* Articles considering periodontitis.

## Appendix A

**Table A1:** Articles matching inclusion criteria. T1DM: type 1 diabetes mellitus; DM: diabetes mellitus; P: periodontitis; CP: chronic periodontitis; SNPs: single nucleotide polymorphisms.

Authors/Year	Conclusions
Hoffman M. et al., 2022 [14]	There are higher sIL-7R serum concentrations at T1DM onset and decreasing levels during therapy, whereas IL-7 was only higher in long-term patients as compared to controls.
Li J. et al., 2022 [15]	Increased serum IL-17A is a risk factor for autoimmune T1DM.
Osman A. E. et al., 2022 [16]	The SNP associated with low production of IL-4 increases the risk of T1DM in young individuals carrying vulnerable HLA alleles/haplotypes.
Ali Y. et al., 2021 [17]	IL-18 gene-promoter polymorphisms might be associated with susceptibility to T1DM in Egyptian children.
El Helaly R.M. et al., 2021 [18]	AA genotype and A allele of IL-10 rs1518111 SNP could be linked to increased risk for T1DM and DM among Egyptian children.
Haghnazari L. et al., 2021 [19]	The G allele of SNP rs1042522 encoding the TP53 gene for IL-6 increases the risk of developing DM in an Iranian population.
Hehenkamp P. et al., 2021 [20]	T1DM monocytes have impaired IL-7 response and lower IL-7R expression.
* Kumar S. et al., 2021 [21]	IL-17A polymorphism was not associated with increased risk for CP in T1DM patients.
Li J. et al., 2021 [22]	The concentration of IL-1β in T1DM patients was significantly higher than that in healthy controls.
Osman A. E. et al., 2021 [22]	IL levels in T1DM were higher than controls.
Tangjittipokin W. et al., 2021 [24]	IL1B SNPs are associated with T1DM susceptibility.
Campos L. P. et al., 2020 [25]	IL-18 polymorphisms were not associated with T1DM onset in children or adults in this population.
Keindl M. et al., 2020 [26]	IL2RA gene variants might increase the risk of developing vascular complications in people with T1DM.
Sharma C. et al., 2020 [27]	IL2-RA gene variants could confer risk alleles for T1DM among the Emirati population.
Boechat-Fernandes A. et al., 2019 [28]	SNP in IL18 gene could be associated with T1DM age onset.
* Borilova Linhartova P. et al., 2019 [29]	Variability in the IL-1B and IL-1RN genes may be one of the factors in the susceptibility to T1DM and P, although the single variants of these polymorphisms are not crucial for protein production.
Campos L. P. et al., 2019 [30]	IL-6 rs1800795 was not associated with adult-onset T1DM. IL-6R rs2228145 was associated with T1DM development in adulthood, and carriers of the minor C allele are at increased risk for adult-onset T1DM.
Lundtoft C. et al., 2019 [31]	IL-7Rα variants may contribute to disease susceptibility against T1DM.
* Borilova Linhartova P. et al., 2018 [9]	CP does not influence the circulating IL-8 levels. Patients with T1DM+CP had higher circulating IL-8 levels than healthy controls+P/non-P.
Seyfarth J. et al., 2018 [32]	Only T1DM children with the protective haplotype had lower IL-7 serum levels.
Al-Lahham Y. et al., 2017 [33]	IL-18 rs187238 was not associated with T1DM in a Euro-Brazilian population.

* Articles considering periodontitis.
